# Combining serum peptide signatures with International Federation of Gynecology and Obstetrics (FIGO) risk score to predict the outcomes of patients with gestational trophoblastic neoplasia (GTN) after first-line chemotherapy

**DOI:** 10.3389/fonc.2022.982806

**Published:** 2022-10-21

**Authors:** Fei Wang, Zi-ran Wang, Xue-song Ding, Hua Yang, Ye Guo, Hao Su, Xi-run Wan, Li-juan Wang, Xiang-yang Jiang, Yan-hua Xu, Feng Chen, Wei Cui, Feng-zhi Feng

**Affiliations:** ^1^ Department of Laboratory Medicine, Peking Union Medical College Hospital, Chinese Academy of Medical Sciences & Peking Union Medical College, Beijing, China; ^2^ Department of Obstetrics and Gynecology, National Clinical Research Center for Obstetric & Gynecologic Diseases, Peking Union Medical College Hospital, Chinese Academy of Medical Sciences & Peking Union Medical College, Beijing, China; ^3^ Department of Gynecological Oncology, Sun Yat-Sen Memorial Hospital of Sun Yat-Sen University, Guangzhou, China; ^4^ Department of Obstetrics and Gynecology, Shanxi Provincial People’s Hospital, Xian, China; ^5^ Department of Obstetrics and Gynecology, Jinan Maternity and Child Health Care Hospital, Jinan, China; ^6^ Department of Clinical Laboratory, State Key Laboratory of Molecular Oncology, National Cancer Center/National Clinical Research Center for Cancer/Cancer Hospital, Chinese Academy of Medical Sciences and Peking Union Medical College, Beijing, China

**Keywords:** gestational trophoblastic neoplasia, serum peptide profiles, machine learning, biomarker, FIGO

## Abstract

**Background:**

Gestational trophoblastic neoplasia (GTN) is a group of clinically rare tumors that develop in the uterus from placental tissue. Currently, its satisfactory curability derives from the timely and accurately classification and refined management for patients. This study aimed to discover biomarkers that could predict the outcomes of GTN patients after first-line chemotherapy.

**Methods:**

A total of 65 GTN patients were included in the study. Patients were divided into the good or poor outcome group and the clinical characteristics of the patients in the two groups were compared. Furthermore, the serum peptide profiles of all patients were uncovered by using weak cation exchange magnetic beads and matrix-assisted laser desorption/ionization time-of-flight mass spectrometry. Feature peaks were identified by three machine learning algorithms and then models were constructed and compared using five machine learning methods. Additionally, liquid chromatography mass spectrometry was used to identify the feature peptides.

**Results:**

Multivariate logistic regression analysis showed that the International Federation of Gynecology and Obstetrics (FIGO) risk score was associated with poor outcomes. Eight feature peaks (*m/z* =1287, 2042, 2862, 2932, 2950, 3240, 3277 and 6626) were selected for model construction and validation by the three algorithms. Based on the panel combining FIGO risk score and peptide serum signatures, the neural network (nnet) model showed promising performance in both the training (AUC=0.9635) and validation (AUC=0.8788) cohorts. Peaks at *m/z* 2042, 2862, 2932, 3240 were identified as the partial sequences of transthyretin, fibrinogen alpha chain (FGA), beta-globin and FGA, respectively.

**Conclusion:**

We combined FIGO risk score and serum peptide signatures using the nnet method to construct the model which can accurately predict outcome of GTN patients after first-line chemotherapy. With this model, patients can be further classified and managed, and those with poor predicted outcomes can be given more attention for developing treatment failure.

## Introduction

Gestational trophoblastic neoplasia (GTN) is a group of clinically rare tumors that develop in the uterus from placental tissue, including invasive mole (IM), choriocarcinoma (CC), placental site trophoblastic tumor (PSTT), and epithelioid trophoblastic tumor (ETT) (1). GTN, which had a poor outcome in the last century, can now have a cure rate of over 90% owing to the combination of different treatment regimens and the refined management for high-risk patients (2). Constant monitoring of serum human chorionic gonadotropin (hCG) levels is essential after GTN treatment and persistently elevated hCG is considered a tumor-marker of poor outcome. The International Federation of Gynecology and Obstetrics (FIGO) scoring system has been developed to predict the prognosis of GTN patients. Based on the FIGO scores, patients are divided into a low-risk group (FIGO score < 7), high-risk group (7 ≤FIGO score ≤ 12), and an ultra-high-risk group (FIGO score > 12) (3). However, more personalized biomarkers are desired to help further evaluate the outcomes of GTN patients after first-line chemotherapy.

The low molecular weight (LMW) peptides in the serum contain abundant histological information, which may be useful in the early diagnosis of disease (4). Notably, specific serum peptide patterns are strongly associated with outcomes of cancer patients (5). However, due to the diverse and complex protein/peptide composition of serum, the fractionation and identification of LMW serum peptides was incredibly challenging. With the development of technology, weak cation exchange magnetic beads (MB-WCX) method has been shown to be efficient in capturing low abundance LMW peptides in serum and matrix-assisted laser desorption/ionization time-of-flight mass spectrometry (MALDI-TOF MS) can be applied to the analysis of captured serum peptides (6). Recently, MALDI-TOF MS-based serum peptide signatures have exhibited enormous potential in identifying patients with early lung cancer (7), cervical intraepithelial neoplasia (8), colorectal cancer (9), esophageal squamous cell carcinoma (10), and coronavirus disease 2019 (COVID-19) (11).

In the present study, the serum peptide profiles of 65 patients with GTN were revealed by MALDI-TOF MS combined with MB-WCX. Simultaneously, this study was dedicated to develop a panel based on combining serum peptide signatures and clinical feature to predict the outcome of GTN patients after first-line chemotherapy using a variety of machine learning models.

## Materials and methods

### Study design and patients population

A prospective collection of peripheral blood of patients with treatment-naïve GTN were conducted within 7 days before the initial therapy at 5 centers from June 2017 to June 2019. Based on FIGO cancer Report and clinical practice in China about management of gestational trophoblastic disease (2, 12–14), single-agent dactinomycin chemotherapy was prescribed as first-line treatment for low-risk GTN (FIGO score of less than 5) and multiagent chemotherapy using 5-FU based regimen or EMACO as first-treatment for low-risk GTN with FIGO score 5-6 or high-risk GTN. Chemotherapy response during first-line therapy was monitored by hCG assay at the start of each treatment cycle. Complete remission (CR) was defined as the normalization of hCG for at least 4 consecutive weeks. Chemotherapy resistance is defined by a plateau in hCG (<10% change) or a rise in hCG over two consecutive cycles, and then second-line chemotherapy is considered. The date cut-off of first-line chemotherapy response ended in completion of first-line chemotherapy for patients who have achieved CR (defined as good outcome group), or for patients who experienced chemotherapy resistance (defined as poor outcome group). And then, the data cut-off of patient survival ended in April 2022. Patients who switched to second-line therapy due to toxicity of first-line chemotherapy were excluded. Additionally, given that first-line treatment regimens for PSTT and ETT differ significantly from that for IM and CC, patients with PSTT or ETT were also excluded.

The patient’s medical records including age, histology, antecedent pregnancy, hCG, FIGO score, FIGO stage, and treatment were collected. This study was approved by Chinese Academy of Medical Science, and Peking Union Medical College (approval no. 18-218/1796). The patients provided their written informed consent in accordance with the declaration of Helsinki.

### Sample collection and preparation

Blood was collected from all patients in the morning and placed at 37°C for 30min to clot. Then, the blood was centrifuged at 3000 rpm for 15 minutes to obtain the serum. The serum of all patients was stored at -80°C for the next step of serum peptide analysis.

### MB-WCX

Serum peptides were extracted using the serum peptide extraction kit (Bioyong Tech, Beijing, China) following the instructions. Firstly, 10μl magnetic beads, 95μl binding buffer and 10μl serum sample were mixed and incubated for 5min at room temperature. Subsequently, the supernatant derived from the mixture by using a magnetic bead separator was added to the wash buffer to remove the high molecular weight peptides/proteins. Finally, the LMW peptides were eluted by adding elution buffer and then subjected to MALDI-TOF MS analysis.

### MALDI-TOF MS

The eluted peptide samples were mixed with 5mg/ml α-cyano-4-hydroxycinnamic acid (CHCA) and 1μl mixture was applied to the MALDI-TOF MS target plate then dried naturally for detection. The MALDI-TOF MS instrument (Bioyong Tech, Beijing, China) was calibrated using commercial peptide and protein calibration standards (Sigma-Aldrich, Louis, MO, USA) before assaying the samples. The spectra were automatically captured in linear mode within the range 1000 -10000 mass-to-change ratio (*m/z*). Each spectrum was normalized, baseline-corrected and smooth-applied using BioExplorer software (Bioyong Tech, Beijing, China). The assay for each sample was repeated three times and the final peak intensity was averaged from the three times.

### Liquid chromatography mass spectrometry

Serum peptides were sequenced and identified by using a nano-liquid chromatography electrospray ionization-tandem mass spectrometry (nano LC/ESI-MS/MS), which consists of an Aquity UPLC system (Waters, USA) and a LTQ Orbitrap XL mass spectrometer (Thermo Fisher Scientific, USA) equipped with a nano-ESI source. Prior to LC-MS analysis of the target samples, the instrument was calibrated using the Pierce Retention Time Calibration Mixture (Thermo Fisher Scientific, USA). The peptides were desalted and then separated by UPLC system and analyzed using MS/MS instrument. The obtained raw mass spectra were analyzed by using Proteome Discoverer software (version 2.1, Thermo Fisher Scientific, USA). The UniProt database (UniProt-*homo*+*sapiens*.fasta, downloaded 10/08/21) was used to perform the identifications. The detailed search parameters were as follows: enzyme: trypsin, max missed cleavages: 2, fixed modifications: Carbamidomethyl, variable modifications: oxidation, protein N-terminal acetylation and deamidation, peptide mass tolerance value: 10ppm, fragment mass tolerance value: 0.02 Da.

### Statistical analysis

All statistical tests were performed using R project (version 4.1.2) and SPSS 20.0 software (SPSS Inc., Chicago, IL, USA). The t-test or Wilcoxon test was used for analysis of continuous data while the chi-square test or Fisher’s exact probability for analysis of categorical data. Progression free survival (PFS) were analyzed using the Kaplan-Meier method and the log-rank test. Multivariate logistic regression analysis was used to identify risk factors for patients with poor outcome. The R packages “caret”, and “nnet” were used for the construction and validation of the models. Receiver operating characteristic (ROC) curves were used to evaluate the diagnostic performance of the model using MedCalc software (version 19.6.1) or Prism GraphPad (version 9.0). *P*<0.05 was considered statistically significant

## Results

### Clinical characteristics and outcomes

Between June 2017 and June 2019, 99 patients’ samples were collected at 5 centers. Thirty-four patients were excluded, including 8 patients with non-GTN, 15 patients with the change of chemotherapy regimen due to toxicity of first-line chemotherapy, 6 patients with PSTT, 1 patient with non-gestational CC, and 4 patients with failure of serum peptide extraction. Ultimately, 65 patients met the eligibility criteria.

**Figure 1:**
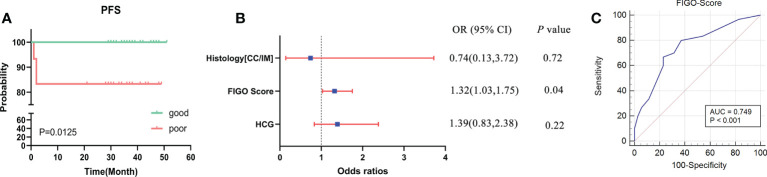
Analysis of the clinical characteristics and outcomes of patients with GTN after first-line chemotherapy **(A)**, disease-free survival (DFS) between two groups with good or poor outcomes; **(B)**, multivariate regression analysis of factors associated with outcomes; **(C)**, ROC analysis using the FIGO risk score to discriminate between different outcome groups.

The clinical characteristics of the 65 GTN patients were summarized in **Table 1**. The median age of all patients was 32 years old, of which 27 were diagnosed with CC and 38 with IM. Patients had a median FIGO score of 3 and 91% (59 of 65) had obvious lesions located in uterus, or other organs including lung, vagina, liver, spleen, great omentum, and pelvic. Based on the status of response during first-line chemotherapy, 35 patients were classified as good outcomes group and 30 patients were classified as poor outcomes group. As shown in **Figure 1A**, patients in the good outcomes group had a longer PFS than those in the poor outcomes group (Hazard Ratio=0.10 (0.02-0.61), *P*=0.0125). Furthermore, patients in the poor outcome group had higher FIGO scores (6 vs 2, *P*<0.001) and higher proportion of CC (57% vs 29%, *P*=0.041) than those in the good outcomes group (**Table 1**). In addition, multivariate logistic regression analysis showed that the FIGO score (Odds Ratio=1.32(1.03-1.75), *P*=0.04) was associated with poor outcomes. (**Figure 1B**). As illustrated in **Figure 1C**, the FIGO risk score exhibited moderate performance in discriminating between patients with different outcomes, with the area under the curve (AUC) of the ROC being 0.749 (*P*<0.001). Overall, the FIGO risk score was effective in predicting outcome of GTN patients after first-line chemotherapy, but its performance could be further strengthened.

**Table 1:** Clinical characteristics and treatments of GTN patients.

Variables	Total (n = 65)	good (n = 35)	poor (n = 30)	*P* value
**Age, Median (Q1, Q3)**	32 (28, 37)	32 (28, 36)	31.5 (27.25, 37.75)	0.707
**hCG, IU/L, Median (Q1, Q3)**	5513 (682, 40831)	2852 (419.06, 10471.5)	28156.5 (2985.25, 75325)	**0.007**
**Lesion location, n (%)**				0.594
Liver/Lung/Spleen/Great omentum	1 (2)	0 (0)	1 (3)	
Lung	13 (20)	8 (23)	5 (17)	
Lung/Pelvic	1 (2)	0 (0)	1 (3)	
Lung/Uterus	20 (31)	12 (34)	8 (27)	
Lung/Vagina/Uterus	1 (2)	0 (0)	1 (3)	
Uterus	21 (32)	9 (26)	12 (40)	
Uterus /Vagina	1 (2)	1 (3)	0 (0)	
Vagina	1 (2)	1 (3)	0 (0)	
None	6 (9)	4 (11)	2 (7)	
**Antecedent pregnancy, n (%)**				0.259
Abortion	10 (15)	5 (14)	5 (17)	
Hydatidiform mole	45 (69)	26 (74)	19 (63)	
Term	10 (15)	4 (11)	6 (20)	
**FIGO Score, Median (Q1, Q3)**	3 (1, 7)	2 (1, 4)	6 (3, 8.75)	**< 0.001**
**FIGO Stage, n (%)**				0.317
I	26 (40)	13 (37)	13 (43)	
II	3 (5)	3 (9)	0 (0)	
III	35 (54)	19 (54)	16 (53)	
IV	1 (2)	0 (0)	1 (3)	
**Histology, n (%)**				**0.041**
CC	27 (42)	10 (29)	17 (57)	
IM	38 (58)	25 (71)	13 (43)	

FIGO, International Federation of Gynecology and Obstetrics; CC, choriocarcinoma; IM, invasive mole.Bold shows the heading of the table or the p-value <0.05.

### Serum peptide profiles analysis

**Figure 2:**
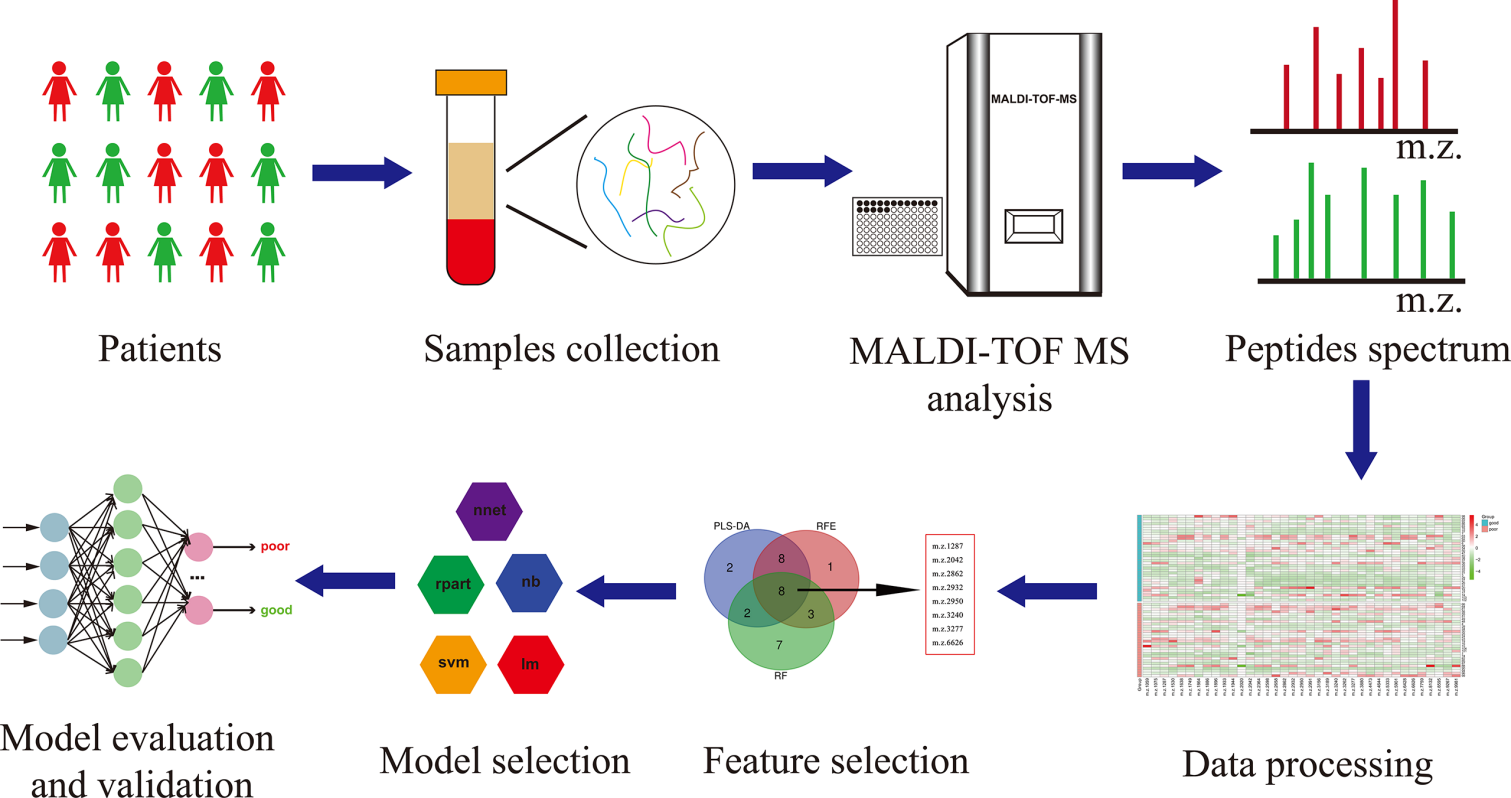
The workflow of serum peptide profiles analysis. Serum from patients containing various peptides/proteins was collected and subsequently processed for analysis using MALDI-TOF MS. The obtained peak patterns were normalized and then the machine learning algorithms were used to perform feature selection. The different machine learning methods were implemented and compared to eventually yield a robust model, which was further evaluated and validated.

**Figure 3:**
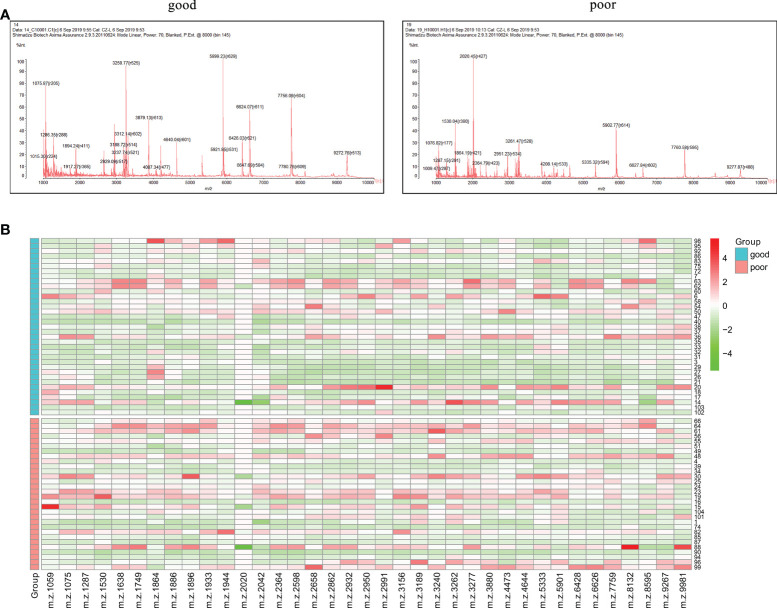
Serum peptide patterns in patients with GTN **(A)**, MALDI-TOF spectra of the serum samples from patients with good or poor outcome; **(B)**, heatmap of the intensity distribution of the 37 peptide peaks for all patients.

The workflow of serum peptide profiles analysis was shown in **Figure 2**. Serum obtained by centrifugation of blood samples from patients contained a wide range of low and high molecular peptides/proteins. LMW serum peptides were extracted by MB-WCX method and analyzed using MALDI-TOF MS. Representative mass spectra of patients with GTN who had good or poor outcome were shown in **Figure 3A**, respectively. A total of 37 peaks in the range of 1000-10000 *m/z* were detected in all 65 patients. The intensities of these peaks are shown in **Figure 3B**, with different patients exhibiting diverse intensity patterns.

### Selection of feature peaks

**Figure 4:**
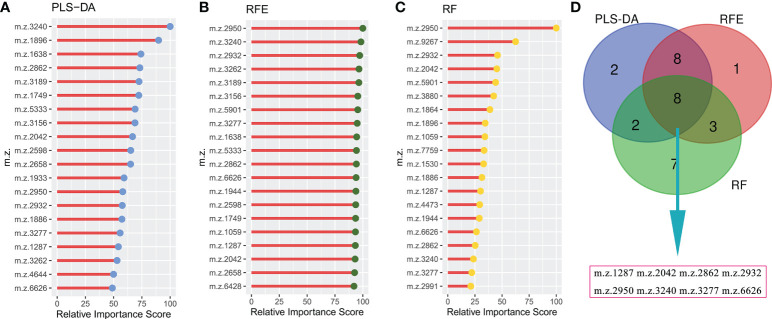
Selecting signature peptide peaks using machine learning algorithms **(A)**, top 20 features prioritized by PLS-DA ranked by the decrease in feature importance scores; **(B)**, top 20 features prioritized by RFE ranked by the decrease in feature importance scores; **(C)**, top 20 features prioritized by RF ranked by the decrease in feature importance scores; **(D)**, final 8 serum peptide features were achieved by taking the intersection of the top 20 features filtered by the three machine learning algorithms.

To construct a robust model for predicting the outcomes of GTN patient, after normalizing the intensity of the peaks, all patients were split into training cohort (n=48) and validation cohort (n=17) with an allocation of 7:3. In training cohort, three machine learning algorithms were used to screen the feature peaks: partial least-squares-discriminant analysis (PLS-DA), recursive feature elimination (RFE) and random forest (RF). All peaks were ranked according to the relative importance score using above three algorithms and the top 20 peaks were shown in **Figures 4A-C**. Furthermore, 8 feature peaks (*m/z* =1287, 2042, 2862, 2932, 2950, 3240, 3277 and 6626) were selected for model construction and validation by taking the intersection of the top 20 peaks filtered by the three algorithms (**Figure 4D**).

### Model construction and validation

**Figure 5:**
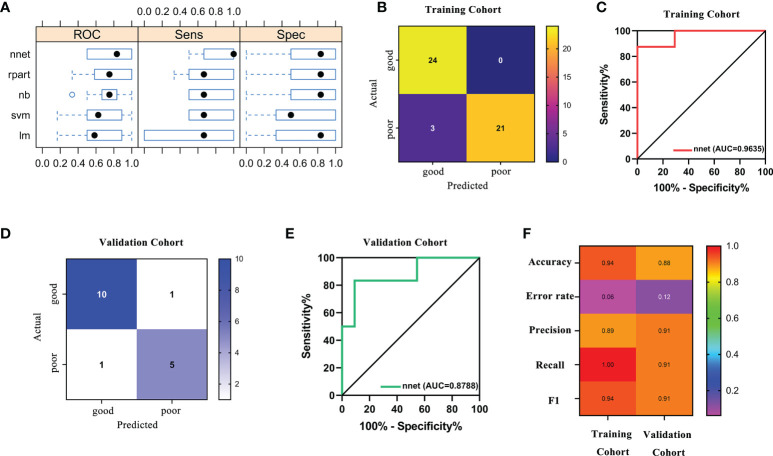
Performance of machine learning models based on FIGO risk scores and serum peptide signatures. **(A)**, comparison of the performance among 5 machine learning methods by cross-validation; **(B)**, confusion matrix of the classification results by the nnet model in training cohort; **(C)**, ROC analysis using the nnet model to discriminate between different outcome groups in training cohort; **(D)**, confusion matrix of the classification results by the nnet model in validation cohort; **(E)**, ROC analysis using the nnet model to discriminate between different outcome groups in validation cohort; **(F)**, summary of accuracy, error rate, precision, recall and F1 for the nnet model in the training and validation cohort.

**Figure 6:**
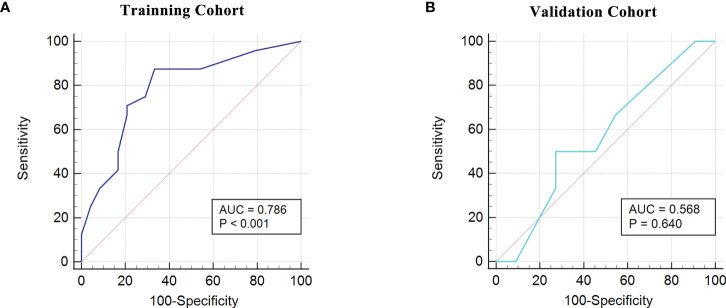
ROC analysis using the FIGO risk score to discriminate between different outcome groups. **(A)**, ROC analysis in the training cohort; **(B)**, ROC analysis in the validation cohort.

Based on the fact that the FIGO score is a vital factor for poor outcome in GTN patients and the eight characteristic peaks that were screened by three machine learning algorithms, we further explored whether these features could be combined to build a powerful model to predict clinical outcomes. Thus, the above features were applied to construct five machine learning model methods, including neural network (nnet), recursive partitioning (rpart), naive bayes (nb), support vector machine (svm), logistic regression model (lm). We compared the performance of five methods by cross-validation to calculate ROC, sensitivity and specificity. As illustrated in **Figure 5A**, the nnet method had higher average ROC, sensitivity and specificity values. Overall, our results indicated the nnet method was superior to the other models; therefore, we chosen the nnet method for further analysis. In the training cohort, the confusion matrix of the nnet method showed that only three patients with poor outcomes were misclassified as good outcomes while no patients with good outcomes were misclassified as poor outcomes (**Figure 5B**). Remarkably, the nnet method reached an AUC of 0.9635 in the training cohort (**Figure 5C**). In the independent validation cohort, the nnet method also achieved a favorable performance: only one patient with good or poor outcome was misclassified respectively, with the AUC value reaching 0.8788 (**Figures 5D, E** **)**. The accuracy, error rate, precision, recall, and F1-score of the nnet method in both the training and validation cohorts were shown in **Figure 5F**. Intriguingly, the AUC for the FIGO score alone to discriminate between good and poor outcome subgroups was 0.786 and 0.568 in the training and validation cohort, respectively (**Figure 6**). Collectively, the nnet machine learning model constructed by combining the FIGO score and serum peptide signatures had satisfactory classification performance in predicting outcomes of GTN patients after first-line chemotherapy.

### Identification of peptide peaks

To further elucidate the role of serum peptide profiles in disease, we used LC-MS/MS for the identification of the peptide peaks. As depicted in **Table 2**, the amino acid sequences of the four peptide peaks were successfully identified. Peaks at *m/z* 2042, 2862, 2932, 3240 were identified as the partial sequences of transthyretin, fibrinogen alpha chain (FGA), beta-globin and FGA, respectively.

**Table 2:** Identified candidate peptide biomarkers.

m/z	Protein sequence	Protein name	Positions in Proteins
1287	NA	NA	NA
**2042**	**ALLSPYSYSTTAVVTNPKE**	**Transthyretin**	**166-184**
**2862**	**MADEAGSEADHEGTHSTKRGHAKSRPV**	**Fibrinogen alpha chain**	**603-629**
**2932**	**KEFTPPVQAAYQKVVAGVANALAHKYH**	**Beta-globin**	**121-147**
2950	NA	NA	NA
**3240**	**SYKMADEAGSEADHEGTHSTKRGHAKSRPV**	**Fibrinogen alpha chain**	**600-629**
3277	NA	NA	NA
6626	NA	NA	NA

NA, not available.Bold shows the parameters of the four peptide peaks.

## Discussion

Currently, most GTN patients could be cured with the preservation of their reproductive function, which benefits from timely and appropriate initial management and prognostic follow-up. It is essential to implement less-toxic monotherapy for patients at low-risk and aggressive multiagent therapy for patients at high-risk, based on the individual risk factors (15). Constant surveillance of hCG levels is imperative and elevated hCG levels after treatment herald poor outcomes, such as relapse or resistance. In this scenario, it would be meaningful to explore individual difference-based biomarkers for predicting treatment outcomes so as to achieve individualized and refined governance for GTN patients after first-line chemotherapy. It is worth noting that the FIGO risk scoring system includes the evaluation of 8-index scale for age, antecedent pregnancy, interval months from index pregnancy, pretreatment serum hCG, largest tumor size, site of metastases, number of metastases and previous failed chemotherapy (15). Previous publications have highlighted the role of the FIGO risk score in assessing prognosis of patients: a higher FIGO score (≥12) forecasted increased probability of treatment failure and poor outcome (16–18). In line with the above studies, we identified the FIGO score as a vital factor for poor outcomes by multivariate logistic regression. Some studies have pointed out that the FIGO risk score is a better predictor of clinical outcomes than single indicators in it (19, 20). On this basis, we attempted to use the ROC curves to determine the power of the FIGO score to predict outcomes and obtained the AUC of 0.749 in our study cohort. It is implied that the FIGO risk score can be used as a valid predictor of outcomes for GTN patients after first-line chemotherapy but there is still room for development.

Proteases are involved in many physiological processes in the body which are crucial for maintaining homeostasis, whilst aberrant protease activity changes are closely associated with tumorigenesis and progression (21). Specifically, proteases can, on the one hand, degrade the extracellular matrix barrier to promote tumor metastasis and progression; on the other hand, cleave proteins in the serum to produce peptide fragments of variable size. In other words, the serum peptide pattern may vary in patients with disparate outcomes. Bedin et al. revealed peculiar changes in the serum peptide profile of colorectal cancer from pre-cancer lesion to metastatic disease, implying the potential usefulness of serum peptide signatures as biomarkers in early diagnosis and prognosis of patients with tumors (22). Therefore, in present study we intended to discover serum peptide signatures that are closely related to the outcome of GTN patients. For all patients, we identified 37 peptide peaks, however not all of them were meaningful. For instance, the peptide peak with *m/z*=2020 had minor variation in most patients. To avoid over-fitting of the constructed model, it was necessary to include only sensible features. Feature selection based on machine learning algorithms has now been reported to filter out risk factors associated with patient diagnosis and prognosis (11, 23). Hereby, we used three machine learning algorithms (PLS-DA, RFE and RF) to help us recognize valuable peptide peaks. PLS-DA is a supervised multivariate classification method that could be a variable selection method by ranking the most important loadings in decreasing order (24). RFE is a greedy algorithm that builds gene sets by iterating continuously, removing the less important genes and selecting the optimal subset from gene sets (25). RF is an algorithm for estimating variable importance based on multiple decision trees, which measures mainly the mean decrease in accuracy or mean decrease in Gini (24). After integrating the three excellent feature selection algorithms, we have determined eight peptide signatures for further analysis.

Recently, it is a hot topic in the field of tumor diagnosis and prognosis evaluation to use selected features in the training set for model building and training by machine learning methods, and to perform the verification in the independent validation set (26–28). In this study, we combined a clinical feature (FIGO score) closely related to outcomes with serum peptide features to generate a prediction panel. Furthermore, models based on this panel were constructed and compared by five machine learning methods (nnet, rpart, nb, svm, lm). Through cross-validation, we found that the neural network (nnet) method outperformed the other methods. The nnet method is considered as a powerful tool for deep learning and artificial intelligence by simulating the functions of the brain’s neural networks to help make decisions (29). The nnet method has been reported to possess enormous strengths in attaining classification of images, and it has been observed to yield a high degree of accuracy in the differentiation of bone marrow cell morphologies (30). She et al. has developed a deep learning survival neural network model which can predict the survival of lung cancer patients and test the reliability of recommended treatments (31). We found that the model constructed using the panel-based nnet method (AUC: 0.9635 in the training cohort; 0.8788 in the validation cohort) showed a substantial improvement in the efficacy of predicting outcomes compared to the FIGO score alone (AUC: 0.786 in the training cohort; 0.568 in the validation cohort).

Machine learning poses a number of challenges when it comes to building accurate models from complicated data, for instance, its complexity and uncertainty make models opaque and difficult to interpret, also known as “black box” (32). On this basis, the selected eight peptides were further identified by using MS/LS-MS to enhance the interpretability of the model. We have determined four of the eight peptides, *m/z* 2042, 2862, 2932, 3240, which were fragments of transthyretin, FGA, beta-globin and FGA, respectively. Transthyretin, also known as prealbumin, is a homotetramer plasma protein of approximately 55 KD that can transport thyroxine by binding to retinol-binding protein (33). It has been reported that transthyretin, a secreted protein downstream of STAT3, could promote oncogenic gene activation, enhance cytokine function in the tumor microenvironment as well as increase the production of reactive oxygen species to facilitate the progression of lung cancer (34). Swiatly et al. reported that transthyretin was a serum peptide biomarker that contributed to the clinical diagnosis of ovarian cancer (35). Moreover, gestational trophoblastic disease is one of the rare causes of hyperthyroidism (36). Thus, it is implied that transthyretin may be closely related to the pathological course and treatment outcome of patients with GTN. Fibrinogen is the precursor of fibrin, which is the key component of the blood coagulation system (37). Fibrinogen alpha chain (FGA) has been reported as a serum peptide signature being a hallmark of a variety of tumors, including colorectal cancer (9), esophageal squamous cell carcinoma (10), non-small cell lung cancer (38), pancreatic ductal adenocarcinoma (39). Duan et al. have revealed FGA as an attractive target for evaluating prognosis in gastric cancer by using DNA microarray analysis (40). Notably, Goldstein et al. found that patients with trophoblastic disease had increased concentration of fibrinogen along with altered fibrinolytic activity (41). In our study, two of the four successfully identified peptides were fragments of FGA, suggesting that FGA may be an important predictor of outcome for GTN patients. Certainly, the underlying mechanism of which deserves further exploration. The combination of the β globin-encoded polypeptides and the α globin-encoded polypeptides could produce distinct hemoglobin tetramers in red blood cells for oxygen transport, while aberrations in this process may lead to the development of severe hemoglobinopathies β-thalassemia (42). For patients with GTN, chemotherapy is a vital treatment, but it can also bring toxicity, such as anemia (43). Given that β globin is a regulator of the maintenance of erythrocyte function, we hypothesized that the toxicity associated with the treatment would have an imprint on it. Therefore, the β globin peptide fragment in the serum may reflect the degree of toxicity of chemotherapy which in turn provides the evidence of outcome.

Overall, the strength of this study was that combining clinical feature and serum peptide signatures constructed a robust model to predict outcomes for GTN patients after first chemotherapy through machine learning approaches. With this model, patients can be further classified and managed, and those with poor predicted outcomes can be given more attention for developing treatment failure or relapse. We were convinced that our study could provide a novel perspective on the treatment and follow-up of GTN after first chemotherapy.

There were also some limitations in this study. The major point was the limited sample size. Nevertheless, GTN as a rare disease has a low incidence. A larger cohort is desired to appraise the reliability of the model in the future. Apart from this, the amino acid sequences of the remaining four serum polypeptide signatures have not been disclosed. Moreover, since there are only 8 differentially expressed peptides identified, these peptides mass should also be verify using multiple reaction monitoring (MRM) based targeted workflow. Peptides along with two MS/MS fragments need to be targeted for absolute quantitation. However, due to the limited volume of serum retained from patients, we may not be able to perform MRM analysis at this time. In the future, we would enroll larger cohorts of patients to establish the level of these peptide during the disease progression by using MRM technology. Notably, other confirmatory experiments could contribute to establishing the utility of these peptides in the prognostic assessment of GTN patients after first-line chemotherapy.

## Data availability statement

The original contributions presented in the study are included in the article/supplementary material. Further inquiries can be directed to the corresponding authors.

## Ethics statement

The studies involving human participants were reviewed and approved by the Ethics Committee of National Cancer Center/Cancer Hospital, Chinese Academy of Medical Science, and Peking Union Medical College. The patients/participants provided their written informed consent to participate in this study.

## Author contributions

WC and F-ZF contributed to the conception and design of this study. HS, X-RW and L-JW contributed to data acquisition. X-SD and X-YJ contributed to data interpretation and analysis. YX and FC contributed to study supervision. Z-RW and FW contributed to manuscript editing. HY and YG contributed to manuscript revising. All authors contributed to manuscript review.

## Funding

This study was supported by the CAMS Innovation Fund for Medical Sciences (CIFMS) (No. 2017-I2M-3-005), Beijing Natural Science Foundation (No. 7202165), National Natural Science Foundation of China (No. 82072361) and the Non-profit Central Research Institute Fund of Chinese Academy of Medical Sciences(2021-PT320-001).

## Conflict of interest

The authors declare that the research was conducted in the absence of any commercial or financial relationships that could be construed as a potential conflict of interest.

## Publisher’s note

All claims expressed in this article are solely those of the authors and do not necessarily represent those of their affiliated organizations, or those of the publisher, the editors and the reviewers. Any product that may be evaluated in this article, or claim that may be made by its manufacturer, is not guaranteed or endorsed by the publisher.
